# Strontium-Doped Bioglass-Laden Gelatin Methacryloyl Hydrogels for Vital Pulp Therapy

**DOI:** 10.3390/jfb15040105

**Published:** 2024-04-17

**Authors:** Sepideh Aminmansour, Ana Beatriz Gomes de Carvalho, Lais Medeiros Cardoso, Caroline Anselmi, Maedeh Rahimnejad, Renan Dal-Fabbro, Erika Benavides, Tiago Moreira Bastos Campos, Alexandre Luiz Souto Borges, Marco C. Bottino

**Affiliations:** 1 Department of Cariology, Restorative Sciences and Endodontics, School of Dentistry, University of Michigan, Ann Arbor, MI 48109, USA; sepideha@umich.edu (S.A.); anab.gomes@hotmail.com (A.B.G.d.C.); lais.cardoso@unesp.br (L.M.C.); caanselm@umich.edu (C.A.); rmaedeh@umich.edu (M.R.); renandf@umich.edu (R.D.-F.); 2 Department of Dental Materials and Prosthodontics, Sao Paulo State University, Sao Jose dos Campos 12245-000, SP, Brazil; alexandre.borges@unesp.br; 3 Department of Dental Materials and Prosthodontics, Sao Paulo State University, Araraquara 14801-903, SP, Brazil; 4 Department of Morphology and Pediatric Dentistry, Sao Paulo State University, Araraquara 14801-903, SP, Brazil; 5 Department of Periodontics and Oral Medicine, School of Dentistry, University of Michigan, Ann Arbor, MI 48109, USA; benavid@umich.edu; 6 Department of Prosthodontics and Periodontology, Sao Paulo University, Bauru 17015-351, SP, Brazil; moreiratiago22@gmail.com; 7 Department of Biomedical Engineering, College of Engineering, University of Michigan, Ann Arbor, MI 48109, USA

**Keywords:** biocompatible materials, bioglass, dental pulp capping, gelatin, hydrogels, strontium

## Abstract

This study aimed to develop gelatin methacryloyl (GelMA)-injectable hydrogels incorporated with 58S bioactive glass/BG-doped with strontium for vital pulp therapy applications. GelMA hydrogels containing 0% (control), 5%, 10%, and 20% BG (*w*/*v*) were prepared. Their morphological and chemical properties were evaluated by scanning electron microscopy/SEM, energy dispersive spectroscopy/EDS, and Fourier transform infrared spectroscopy/FTIR (n = 3). Their swelling capacity and degradation ratio were also measured (n = 4). Cell viability (n = 8), mineralized matrix formation, cell adhesion, and spreading (n = 6) on DPSCs were evaluated. Data were analyzed using ANOVA/post hoc tests (α = 5%). SEM and EDS characterization confirmed the incorporation of BG particles into the hydrogel matrix, showing GelMA’s (C, O) and BG’s (Si, Cl, Na, Sr) chemical elements. FTIR revealed the main chemical groups of GelMA and BG, as ~1000 cm^−1^ corresponds to Si-O and ~1440 cm^−1^ to C-H. All the formulations were degraded by day 12, with a lower degradation ratio observed for GelMA+BG20%. Increasing the concentration of BG resulted in a lower mass swelling ratio. Biologically, all the groups were compatible with cells (*p* > 0.6196), and cell adhesion increased over time, irrespective of BG concentration, indicating great biocompatibility. GelMA+BG5% demonstrated a higher deposition of mineral nodules over 21 days (*p* < 0.0001), evidencing the osteogenic potential of hydrogels. GelMA hydrogels incorporated with BG present great cytocompatibility, support cell adhesion, and have a clinically relevant degradation profile and suitable mineralization potential, supporting their therapeutic potential as promising biomaterials for pulp capping.

## 1. Introduction

The primary cause of dental pulp infection is cariogenic bacteria that colonize and form biofilm on dental tissues, resulting in inflammation and further tissue necrosis [1,2,3]. The traditional treatment for infected dental pulp is root canal therapy (RCT), which involves removing all the pulp tissue, cleaning and shaping the infected area, and then filling it with inert materials [4,5]. However, RCT presents some drawbacks, such as weakness of reminiscent tooth structure, leading to brittleness, a loss of biological defense mechanisms, tooth discoloration, and others [5,6].

Recent studies have highlighted the efficacy of direct vital pulp procedures, such as pulp capping, as a less invasive dental treatment than root canal therapy (RCT). These approaches aim to sustain pulp vitality and potentially restore damaged dentin tissue [7]. Notably, significant advances have been made in developing biomaterials for pulp capping that can influence the human immune system, thereby steering a targeted regenerative outcome [8]. However, when considering the ideal properties of a hydrogel for tissue regeneration (i.e., injectability, proper mechanical properties and degradation profile, and cytocompatibility) and the complexity of the dentin/pulp complex [9], there is still space for exploring new natural and synthetic biomaterials that might induce dentin/pulp complex tissue regeneration [10].

When considering natural polymers, gelatin-based biomaterials are highlighted as an excellent alternative for tissue engineering applications [9]. In this context, by modifying gelatin, it is possible to obtain gelatin methacryloyl (GelMA), a semi-synthetic polymer that modifies amine-containing side groups of gelatin (Gel) with methacrylamide and methacrylate groups [11]. It presents several beneficial properties for tissue regeneration, such as cytocompatibility, hydrophilicity, and a suitable degradation rate when exposed to metalloproteinases [12]. Another great advantage of this material is the possibility of making GelMA hydrogels, which can fulfill variable and complex anatomies through their injectability property [13,14]. Furthermore, GelMA hydrogels can be efficiently mixed with other components such as nanotubes, antibiotics, or particles (i.e., bioglass) [11].

Bioactive glass, commonly called bioglass (BG), is a biomaterial known for its biocompatibility and unique dual properties of being osteoconductive and osteoinductive. Additionally, it facilitates the differentiation of dental pulp stem cells (DPSCs) into odontogenic cells and promotes the formation of blood vessels in endothelial cells [15]. Mainly, 58S BG has demonstrated excellent biocompatibility and bioactivity by inducing mineral formation and cell differentiation [16]. Moreover, 58S BG containing chlorine presents exceptional potential for tissue engineering applications since this chemical component is naturally present in human organisms [17]. BGs can be doped with ions such as strontium (Sr), which stimulates osteoblasts [18] and induces odontogenesis and angiogenesis [19], evidencing their great bioactive potential in repairing the dentin/pulp complex [20].

In this context, the present study aimed to develop GelMA hydrogels modified with varying concentrations of 58S BG doped with Sr ions, a biomaterial designed for minimally invasive endodontic procedures. Combining the benefits of this polymer and the excellent bioactivity demonstrated by BG, we hypothesize that these new injectable and incorporated hydrogels might be a promising biomaterial for vital pulp therapy treatments.

## 2. Materials and Methods

### 2.1. Bioglass Synthesis

Bioglass (BG) was produced via the sol–gel route, following a methodology described previously [21]. Firstly, silicic acid (H_4_SiO_4_) was used as a silica precursor; for that, sodium metasilicate (Na_2_SiO_3_·5H_2_O) was passed through an aqueous solution (10% *w*/*w*) with an ion exchange resin, forming silica sol through the exchange of Na^+^ and H^+^ ions. Then, this solution was filtered with a vacuum pump. The concentration of silicic acid was determined by titrating this component with standardized sodium hydroxide from which it was obtained, so the final concentration was 60% H_4_SiO_4,_ 36% CaCl_2_, and 4% NH_4_H_2_PO_4_. After mixing all the components, the solution was dried in an oven for 12 h at 100 °C, and the calcination process was conducted for 5 h at 500 °C. At the end of this process, strontium chloride (10% *w*/*w*) was added to the composition. Finally, the bioglass was crushed and sieved using a 375 mesh (75 μm).

### 2.2. Gelatin Methacryloyl Synthesis

GelMA synthesis was prepared following previous methodologies [11,22]. Type-A Gel (10% *w*/*v*) was dissolved in phosphate-buffered saline (PBS; Gibco™, Thermo Fisher Scientific, Waltham, MA, USA) on a heating plate at 50 °C and constantly stirred. Then, 8 mL of methacrylate was slowly added. After 2 h, 8 mL of PBS (Gibco™, Thermo Fisher Scientific, Waltham, MA, USA) was added to the solution to interrupt the methacrylate reaction. After that, dialyze membranes (12–14 kDa) were filled with the solution and immersed in deionized water (DI) at 45 ± 5 °C for one week, changing the water twice daily. After the dialysis process, the solution was filtered, placed in 50 mL falcon tubes, frozen at −80 °C overnight, and lyophilized (Labconco FreeZone 2.5 L, Labconco Corporation, Kansas City, MO, USA) for seven days, and the final GelMA foam was stored at −20 °C [12].

To prepare the hydrogels, a 15% GelMA solution was made using 450 mg of GelMA foam and 3 mL of PBS (Gibco™, Thermo Fisher Scientific, Waltham, MA, USA). The two components were mixed and carried on a heating plate at 50 ± 5 °C with 300 rpm stirring. Then, 5%, 10%, and 20% of BG powder (*w*/*v*) were added to the GelMA solution and mixed under the same conditions. One group of pure GelMA (without any percentage of BG) was considered the control group. After the proper dispersal of BG in GelMA, silicon (CutterSil Putty PLUS, Kulzer US, South Bend, IN, USA) molds (6 × 2 or 8 × 3 mm) were filled with the solutions (60 and 150 μL, respectively) and cross-linked using a light cure (Bluephase Style, Ivoclar, Amherst, NY, USA) for 15 s for pure GelMA, GelMA+BG 5%, and GelMA+BG 10% and 30 s for GelMA+BG 20% on each side.

### 2.3. Scanning Electron Microscopy and Energy Dispersive Spectroscopy

The microstructure of the pure and incorporated hydrogels was analyzed under scanning electron microscopy (SEM) and energy dispersive spectroscopy (EDS) (Tescan MIRA3 FEG-SEM, Tescan USA Inc., Warrendale, PA, USA). The hydrogels (n = 3/group) were prepared and freeze-dried in the lyophilizer for 24 h. Then, the hydrogels were cross-sectioned to expose their internal microstructure and gold-sputtered for 90 s.

### 2.4. RAMAN Spectroscopy

The RAMAN spectrum was produced to assess information about the silicate structures of the material [23], with it being possible to observe the formation of non-bridged bonds (NBOs), confirm if the structure of the glass silicate formed in the material was similar to the expected structure for 58S BG, and verify if the incorporation of Sr ions was successful in the material’s network. The spectrum was collected using the LabRam HR Evolution spectrometer (HORIBA Scientific, Kyoto, Japan) grouped to an optical microscope with an Nd: Yag laser at 532 nm (laser power 100%), in a spectrum range of 200–1200 cm^−1^; three scans were taken with an acquisition time of 30 s and resolution of 600 µm.

### 2.5. Fourier Transform Infrared Spectroscopy

Fourier transform infrared spectroscopy (ATR-FTIR, Nicolet iS50, Thermo Fisher Scientific, Inc., Waltham, MA, USA) was performed to analyze the chemical stretches presented by GelMA and GelMA incorporated with BG groups. Sixteen scans were collected with a spectrum between 4000 and 500 cm^−1^ and a resolution of 4 using a diamond crystal. Baseline correction spectra were then centered and normalized for analysis.

### 2.6. Swelling Capacity

To assess the swelling capacity of the hydrogels (6 × 2 mm, n = 3/group), they were immersed in vials containing 5 mL of PBS (Gibco™, Thermo Fisher Scientific, Waltham, MA, USA) and stored at 37 °C for 24 h. Subsequently, the hydrogels were gently blotted dry using tissue paper (Kimwipes Kimtech, Kimberly-Clark Professional, Irving, TX, USA), and their wet weights (*W_w_*) were measured using an analytical balance. Then, the same hydrogels were lyophilized for 24 h and weighed again to determine their dry weights (*W_d_*). The *swelling* capacity (%) was calculated according to the following formula:

$$Swelling\;ratio\;\left(\%\right)=\frac{\left(W_{w}-W_{d}\right)}{W_{w}}\times 100$$

### 2.7. Degradation Ratio

Hydrogels (6 × 2 mm, n = 4/group) were placed into glass vials (VWR International, LLC, Radnor, PA, USA) containing 5 mL of PBS (Gibco™, Thermo Fisher Scientific, Waltham, MA, USA) and 1 U/mL of collagenase type A at 37 °C. The initial weight (*W*_0_) was measured using an analytical balance, and every two days (*W_t_*), the hydrogels were removed from the vials, washed 2 times with DI water, blot-dried with paper tissue, and weighed again. PBS–collagenase (Gibco™, Thermo Fisher Scientific, Waltham, MA, USA) was replaced at each time point to keep the enzyme active during the test. This process was repeated until the hydrogels were wholly dissolved. The *degradation ratio* (%) was calculated according to the following formula:

$$Degradation\;ratio\;\left(\%\right)=\;\frac{W_{t}}{W_{0}}\times 100$$

### 2.8. Mechanical Test

To evaluate the mechanical properties, the hydrogels (8 × 3 mm, n = 8/group) were subjected to compressive stress under dry and wet conditions. Regarding the dry condition, the hydrogels were prepared and tested immediately after cross-linking. For the wet condition, they were stored in PBS (Gibco™, Thermo Fisher Scientific, Waltham, MA, USA) at 37 °C for 24 h, blot-dried, and then tested. The compression test was performed using a mechanical testing machine (MTESTQuattro, ADMET Inc., Norwood, MA, USA) at a 1 mm/min strain rate and a cell load of 250 kgf until 30% of the hydrogel’s deformation was achieved. Then, the software (MTESTQuattro-PC-based Controller version 5.07.13, ADMET Inc., Norwood, MA, USA) linked to the testing machine calculated the modulus of elasticity using the stress/strain curve.

### 2.9. Cell Viability

For all biological analysis, dental pulp stem cells (DPSCs) isolated from adult third molars were purchased from Lonza Bioscience (PT-5025, Lonza Bioscience, Walkersville, MD, USA), and cells at passages 5–7 were used [24]. The experimental in vitro assay was conducted for two calibrated operators in duplicate. To assess the cell toxicity of the hydrogels, 6 × 2 mm specimens (n = 8/group) were prepared. UV light was used on each side for 30 min to promote the disinfection of the hydrogel. After that, they were individually placed into sterile glass vials containing 5 mL of α-MEM media supplemented with 10% FBS, L-glutamine, 1% penicillin–streptomycin, and 1 U/mL of collagenase type A. Hydrogels were incubated at 37 °C. According to each time point (1, 3, and 7 days), 500 μL of aliquots were collected to be used for the cell viability assay. After collecting the aliquots, an equal amount of fresh collagenase medium was added to the vials.

DPSCs were seeded (3 × 10^3^ cells/well) and allowed to adhere to the bottom of the wells on a 96-well plate for 24 h at 37 °C and 5% of the CO_2_ environment. After that, the collected aliquots were filtered using a 0.22 μm membrane, and the cells’ media were replaced with 100 μL of the aliquots, which were left in contact with the cells for 24 h.

After 24 h, the aliquots were replaced with 100 μL of 10% Alamar Blue (Invitrogen, Carlsbad, CA, USA) solution and allowed to react for 3 h at 37 °C in a 5% CO_2_ environment. The fluorescence intensity at 530 and 590 nm was analyzed (SpectraMax iD3, Molecular Devices, LLC, San Jose, CA, USA) against a blank column. Cells on a plate with α-MEM media were considered the negative control (NC), and phenol solution 0.3% (Reagents, Charlotte, NC, USA) was considered the positive control (PC). The fluorescence intensity values were converted to percentages and compared to the NC to determine the cell viability at each time point.

### 2.10. Mineralized Nodule Formation

Mineral deposition was evaluated at 14 and 21 days in osteogenic media. DPSCs at a density of 1 × 10^4^ were seeded on top of hydrogels (n = 6/group) and incubated according to each time point. Then, hydrogels were fixed with 70% ethanol for 1 h at 4 °C, washed with distilled water, and incubated with Alizarin Red staining (40 mM; pH 4.2) for 20 min. After staining, hydrogels were washed with distilled water ten times. Then, nodules were dissolved in hexadecylpyridinium chloride monohydrate (10%; Sigma Aldrich, St. Louis, MO, USA), and the absorbance was read at 570 nm (SpectraMax iD3, Molecular Devices, LLC, San Jose, CA, USA). Hydrogels without cells were used as a background control.

### 2.11. Cell Adhesion (Fluorescence)

To evaluate the cell adhesion and spreading on the hydrogels, 6 × 2 mm (n = 4/group) specimens were made, and DPSCs (1 × 10^4^ cells/well) were seeded on top of the hydrogels. After seeding, cells were fixed using 4% paraformaldehyde (Sigma Aldrich, St. Louis, MO, USA) at 12, 24, and 48 h. Then, hydrogels were washed with PBS (Gibco™, Thermo Fisher Scientific, Waltham, MA, USA) and stained with a red fluorescent probe for actin filaments (1:20; ActinRed 555 ReadyProbes reagent; Invitrogen) for 30 min or with a blue fluorescent staining DAPI (1:5000; Thermo Fisher Scientific, Waltham, MA, USA) for DNA/nuclei. Finally, hydrogel surfaces were analyzed at 10× magnification using a fluorescence microscope (ECHO Revolve Microscope; Discover Echo Inc., San Diego, CA, USA).

### 2.12. Statistical Analysis

After collecting the results, normality and homoscedasticity were assumed by Shapiro–Wilk and Levene; thus, ANOVA and post hoc tests were selected for data analysis (α = 0.05). The GraphPad PRISM computer program (GraphPad Software version 10.2.1, San Diego, CA, USA) was used for statistical testing.

## 3. Results and Discussion

### 3.1. SEM and EDS

**Figure 1:**
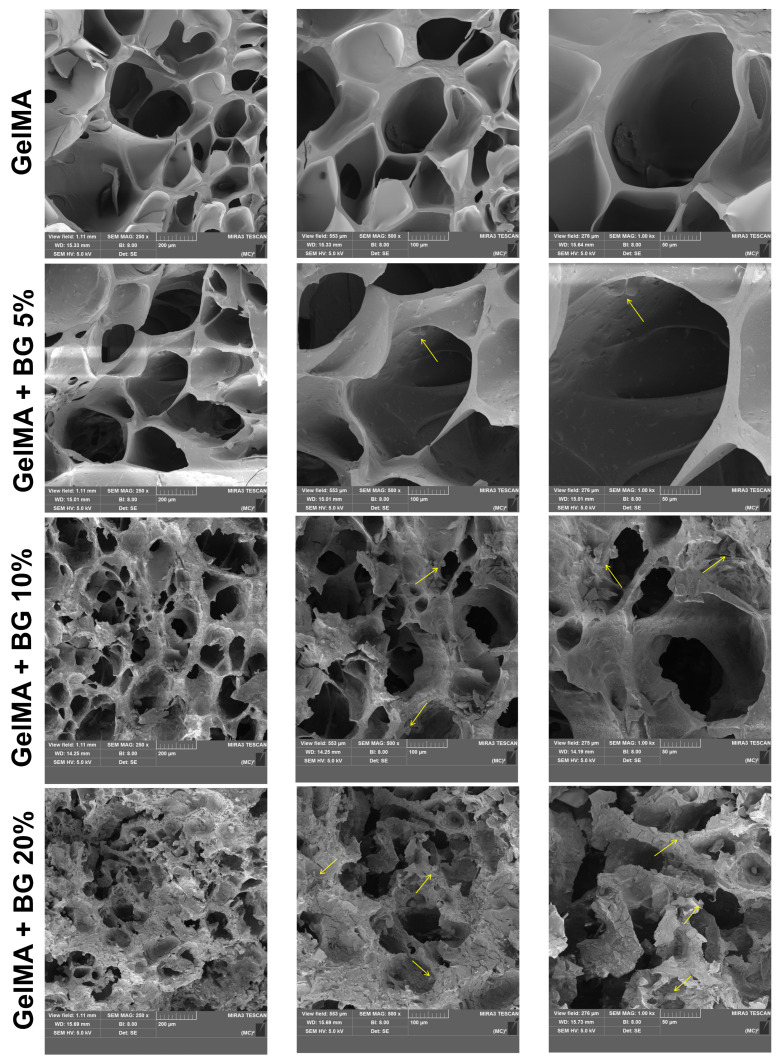
Morphological characterization by SEM pictures at 250×, 500×, and 1 k× magnifications of GelMA hydrogels incorporated with varying BG concentrations. Yellow arrows show the BG particles dispersed inside the hydrogel matrix.

**Figure 2:**
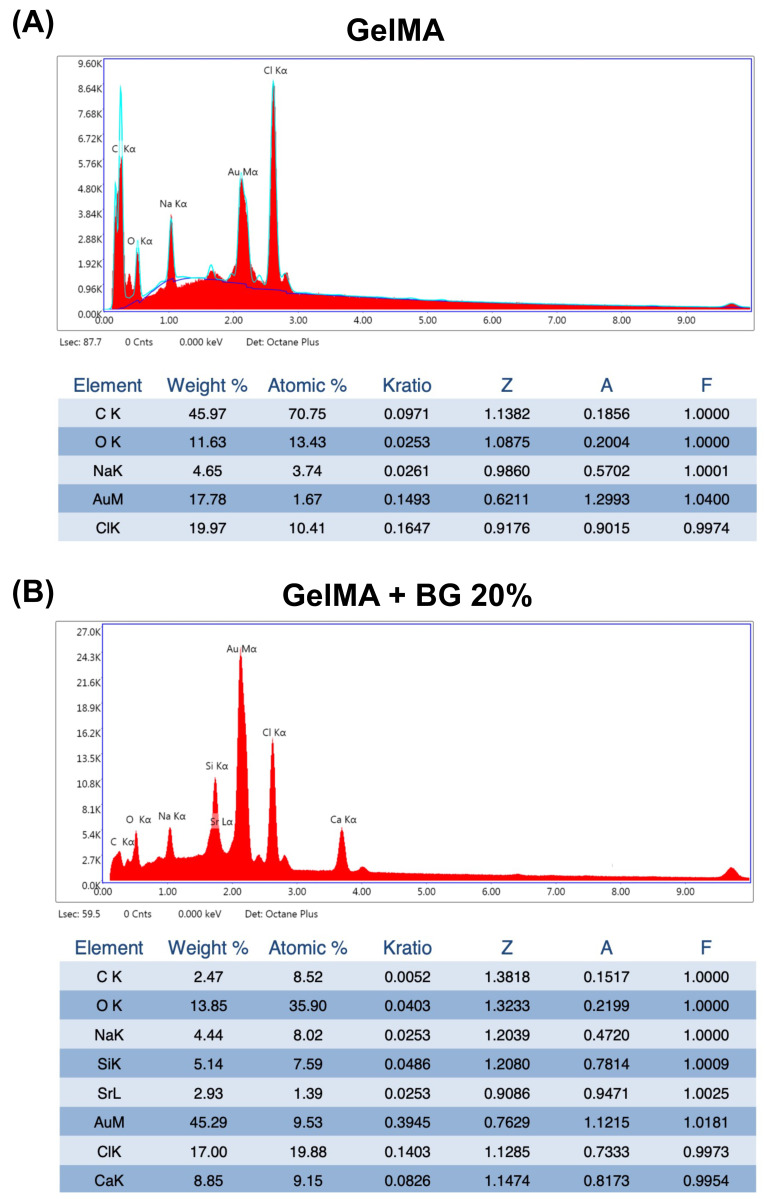
(**A**) Chemical characterization by EDS spectrum of GelMA hydrogels and (**B**) chemical characterization by EDS spectrum of GelMA+BG 20%.

Representative SEM images of all the analyzed groups are shown in Figure 1. The porous honeycomb-like structure typical of hydrogels can be observed in all the groups, and diamond-shaped crystals can be found dispersed in the polymeric matrix (yellow arrows), suggesting the presence of BG particles. The concentration of BG directly correlates with the increased presence of these particles without disrupting the porous structure of the hydrogels. This evidence is confirmed by the EDS spectra, which showed the chemical components of pure GelMA as C, O, Na, and Cl (Figure 2A) and GelMA+BG 20% as Si, Cl, and Na alongside the natural components of pure GelMA (Figure 2B).

Various compositions and manufacturing techniques for BG have been extensively documented in the literature, aiming to produce particles characterized by exceptional chemical stability and biocompatibility [21,25,26]. In this study, 58S BG was synthesized via the sol–gel method, incorporating chlorine into its composition, an element verified by EDS spectra. These modifications to the BG composition are promising to foster enhanced biological interactions between hydrogels and cells [26], a topic that will be illustrated in subsequent discussions.

### 3.2. FTIR

**Figure 3:**
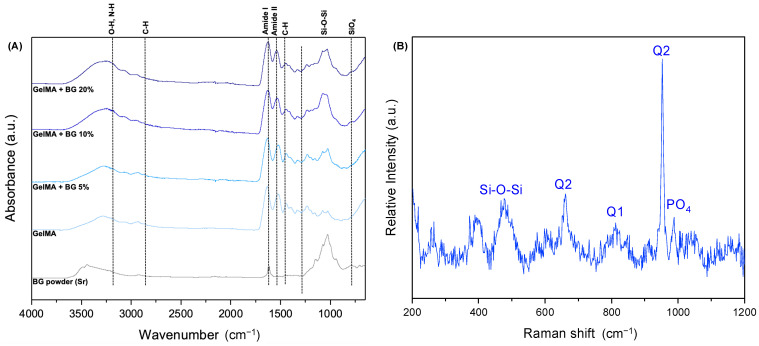
(**A**) Fourier transform infrared spectroscopy graph according to the different concentrations of BG incorporated into GelMA and 58S BG powder. (**B**) RAMAN spectrum for BG powder.

The FTIR spectra (Figure 3A) exhibited distinctive peaks corresponding to GelMA, notably N-H and OH stretches within the 3000–3500 cm^−1^ range. Also, characteristic amide I and II groups were discernible around 1650 and 1600 cm^−1^. At 1440 cm^−1^, a noticeable C-H stretch was observed. Within the spectra, the Si-O-Si-bridged oxygen bonds, a hallmark of BG, were expressed within the range of 1000–1030 cm^−1^, showing an increasing intensity relative to the concentration of BG. These stretches indicate that Si might be linked with different components, such as -OH or Ca and Sr ions. Alongside the Si-O-Si stretch, the presence of the silica tetrahedron SiO_4_ was evident at 700–850 cm^−1^.

### 3.3. RAMAN Spectrum

The RAMAN spectrum (Figure 3B) distinctly highlights the Q″ bands, which indicate the silica tetrahedron and its associated chemical bonds. Each number related to these bands indicates the average number of bridging bond atoms per silicon, ranging from 0 to 4 [27]. Precisely, the Q1 band corresponds to an increase in non-bridging bonds (NBO), denoting the Si-O-X stretch, where X″ could represent network modifier elements like Ca, Na, or therapeutic ions such as Sr [28,29]. Upon analyzing the RAMAN spectrum, indications strongly suggest that the Q1 band around 800 cm^−1^ potentially relates to Sr, suggesting its incorporation within the BG structure [21]. Also, the Q2 band around 700 cm^−1^ indicates the possibility of a silicon atom linked to strontium and calcium at the same silica tetrahedron, creating an asymmetric structure. Additionally, the presence of Q1 and Q2 bands indicates the BG’s high bioactivity, implying a silicon atom with two free sites for interaction with other atoms.

### 3.4. Mechanical Test

**Figure 4:**
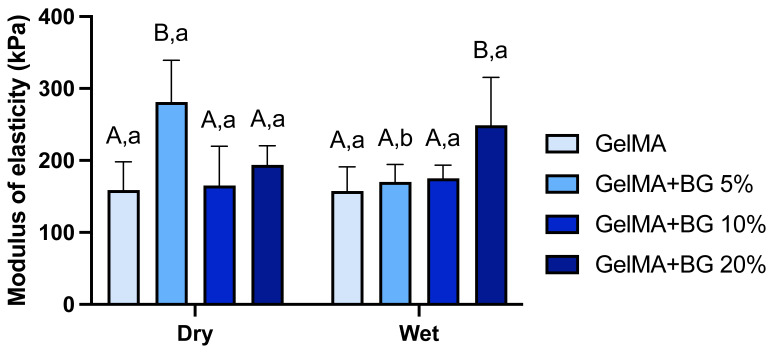
Mechanical properties of GelMA- and BG-incorporated hydrogels. Results are presented as mean and SD. Different capital letters indicate significant differences between groups within each condition. Different lower-case letters demonstrate significant differences between conditions within each group. Two-way ANOVA/Sidak’s post hoc test (n = 8; α = 0.05).

The evaluation of the mechanical properties of biomaterials is of great importance to the tissue engineering field to ascertain if the developed hydrogels can mimic the mechanical behavior of natural tissues and consequently provide the required support when placed in the desired area. Figure 4 reveals that GelMA+BG 5% exhibited a superior modulus of elasticity in dry conditions, while GelMA+BG 20% outperformed all the groups in wet conditions. When comparing the conditions within the groups, only GelMA+BG 5% presented a significant difference. The pure GelMA hydrogels presented an average of ~160 kPA for both conditions, which agrees with previous studies published by our research group, and the presence of BG particles has been shown to be a determinant factor in influencing the mechanical properties of GelMA-based hydrogels. One possible explanation for improving the mechanical properties of BG-incorporated hydrogels is the chemical interactions between the particles and hydrogel matrix, promoting a greater linking potential [30].

### 3.5. Swelling and Degradation

**Figure 5:**
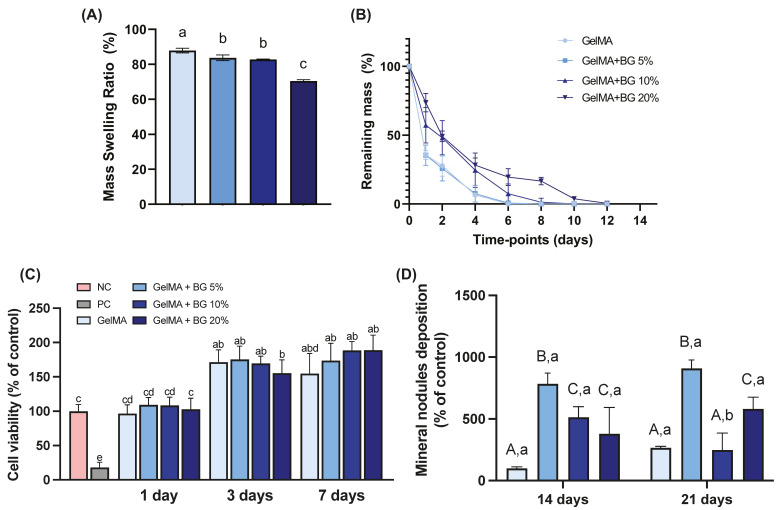
(**A**) Mass swelling ratio (%) for GelMA and different concentrations of BG. ANOVA/Games–Howell (n = 3; α = 0.05); (**B**) remaining mass (%) for hydrogels after different time points. Results are presented as mean and confidence interval (CI). (**C**) Cell viability (% of control) after 1, 3, and 7 days. The percentage of cell viability was normalized by the fluorescence of the negative control (NC) group. Welch’s ANOVA/Games–Howell (n = 8; α = 0.05). (**D**) Mineralized matrix formation (% of control for each period). Two-way ANOVA/Sidak’s post hoc test (n = 6; α = 0.05). Results are presented as mean and SD for bar graphs. Different capital letters indicate significant differences between groups within each time point. Different lower-case letters demonstrate significant differences between time points within each group.

The analysis of the mass swelling ratio of the hydrogels after 24 h (Figure 5A) reveals an inverse relationship between the swelling capacity and the concentration of BG within the hydrogels. The swelling capacity of pristine GelMA was 87.92 ± 1.33%. For GelMA mixed with BG at 5%, the swelling capacity was 83.68 ± 1.64%; for 10%, it was 82.81 ± 0.26%, and for 20%, it was 70.45 ± 0.78%. There was no significant difference in swelling capacity between the 5% and 10% concentrations. The swelling capacity of hydrogels is directly related to their hygroscopicity, and a higher swelling ratio usually indicates a microstructure with large pores, which, when in contact with aqueous media, will accelerate the degradation rate of the material [11]. When a higher concentration of BG was added to the polymeric matrix (i.e., BG 20%), the hydrogels’ microstructure suffered an alteration, possibly reducing the size of the pores and consequently reducing the swelling capacity of the hydrogels.

Figure 5B illustrates the remaining mass of the hydrogels during the degradation test across various time points. Interestingly, higher concentrations of BG resulted in a slower degradation profile compared to both pristine GelMA and GelMA+BG 5%. However, it is noteworthy that, despite this disparity, all the hydrogel variations underwent complete degradation within 12 days. The degradation ratio can be correlated with the Alizarin Red results (Figure 5D) since the degradation of hydrogels occurred concomitantly to mineral nodule deposition, which is one of the desirable properties for a biomaterial when considering dentin/pulp complex regeneration [10]. This indicates that calcified tissue forms at the interface between the hydrogel and adjacent tissue.

### 3.6. Cell Viability

The cell viability depicted in Figure 5C indicates that on day 1, all the hydrogels exhibited cytocompatibility statistically similar to the negative control (NC), consisting of cells and growth media. By days 3 and 7, all the groups displayed statistically similar cell viability. Notably, there was an increase in cell viability across all the groups compared to the initial time point. These findings indicate robust cytocompatibility across all the groups (*p* ≥ 0.6196), with the average cell viability exceeding 70%, typically serving as the threshold for cell viability assessments.

One of the most significant advantages of the 58S BG doped with Sr ions used in this study is the chlorine in its chemical composition, which led to great biocompatibility since chlorine is a natural chemical element found in the human body [17]. All concentrations of BG incorporated into the GelMA hydrogels analyzed in this study presented a cell-friendly behavior, confirming the cytocompatibility of chlorinated 58S BG, which was previously compared to non-chlorinated BGs [26].

### 3.7. Mineralized Matrix Formation

The results obtained from the Alizarin Red assay for mineral nodule formation (as shown in Figure 5D) indicate that after 14 days, both GelMA+BG 10% and GelMA+BG 20% had similar levels of mineral formation, which were higher than pristine GelMA. After 21 days, GelMA+BG 10% showed a decrease in mineral formation, while the concentrations of 0% and 20% remained stable. Regardless of the time point (14 or 21 days), a concentration of 5% BG was found to be the most effective in terms of mineral production.

According to the literature, Sr ions play an essential role in the bioactivity of biomaterials since it was previously reported that the presence of Sr leads to a more significant odontogenesis potential of DPSCs in vitro and a higher dentin volume and density in vivo [31]. Our findings showed that the Sr-doped BG hydrogels increased the mineral matrix formation after 14 and 21 days, regardless of the concentration, compared to the pristine GelMA hydrogels. Besides Sr ions, the chlorine in BG’s chemical composition also favors the formation of apatite-like phases [21]. Considering biocompatibility and bioactivity, the concentration of 5% of BG in GelMA hydrogels demonstrated higher cell viability and mineral matrix formation.

### 3.8. Cell Adhesion and Spreading

**Figure 6:**
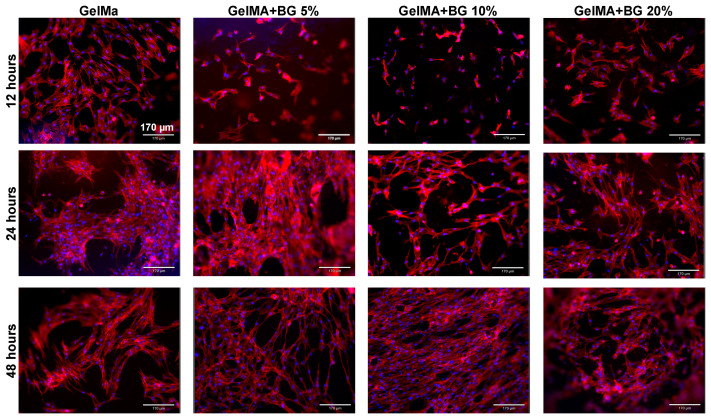
Cell adhesion and spreading for the analyzed groups for different time points (12, 24, and 48 h). Actin filaments (red) were stained with ActinRed 555 reagent, and cell nuclei (blue) were labeled with DAPI.

The cell adhesion and spreading were evaluated by an F-actin assay, in which the cell nuclei were stained in blue and the cytoskeleton was stained in red, as shown in Figure 6. After 12, 24, and 48 h of culture, it was possible to observe by a qualitative analysis that the presence of BG is not favorable for cell attachment at the earliest time point. When observing the pictures corresponding to 12 h, it is possible to observe that the cells are less elongated and spread out in the BG-laden groups. However, after 24 and 48 h, the cells are spread adequately at the hydrogels’ surface, regardless of the group. Our findings agree with the literature that has evaluated the influence of Sr-doped BGs through a cement approach, confirming the suitable cell attachment of DPSCs at later time points [31].

## 4. Conclusions

Gelatin methacryloyl hydrogels incorporated with 58S BG doped with Sr ions present suitable cytocompatibility, support cell adhesion, and have a clinically relevant degradation profile and mineralization properties, suggesting their therapeutic potential for pulp capping.

## Data Availability

All data and materials are available on request from the corresponding author. The data are not publicly available due to ongoing research using a part of the data.
